# Sheep as a large animal model for hearing research: comparison to common laboratory animals and humans

**DOI:** 10.1186/s42826-023-00182-3

**Published:** 2023-11-27

**Authors:** Po-Yi Lue, Mark H. Oliver, Michel Neeff, Peter R. Thorne, Haruna Suzuki-Kerr

**Affiliations:** 1 Department of Physiology, The University of Auckland, Auckland, New Zealand; 2 Eisdell Moore Centre, The University of Auckland, Auckland, New Zealand; 3 Liggins Institute, The University of Auckland, Auckland, New Zealand; 4 Ngapouri Research Farm Laboratory, University of Auckland, Waiotapu, New Zealand; 5 Department of Surgery, Auckland District Health Board, Auckland, New Zealand; 6 Section of Audiology, The University of Auckland, Auckland, New Zealand

**Keywords:** Hearing, Sensorineural hearing loss, Cochlea, Inner ear, Animal model, Cochlear implant, Sheep, Auditory neuroscience, Large animal

## Abstract

Sensorineural hearing loss (SNHL), caused by pathology in the cochlea, is the most common type of hearing loss in humans. It is generally irreversible with very few effective pharmacological treatments available to prevent the degenerative changes or minimise the impact. Part of this has been attributed to difficulty of translating “proof-of-concept” for novel treatments established in small animal models to human therapies. There is an increasing interest in the use of sheep as a large animal model. In this article, we review the small and large animal models used in pre-clinical hearing research such as mice, rats, chinchilla, guinea pig, rabbit, cat, monkey, dog, pig, and sheep to humans, and compare the physiology, inner ear anatomy, and some of their use as model systems for SNHL, including cochlear implantation surgeries. Sheep have similar cochlear anatomy, auditory threshold, neonatal auditory system development, adult and infant body size, and number of birth as humans. Based on these comparisons, we suggest that sheep are well-suited as a potential translational animal model that bridges the gap between rodent model research to the clinical use in humans. This is especially in areas looking at changes across the life-course or in specific areas of experimental investigation such as cochlear implantation and other surgical procedures, biomedical device development and age-related sensorineural hearing loss research. Combined use of small animals for research that require higher throughput and genetic modification and large animals for medical translation could greatly accelerate the overall translation of basic research in the field of auditory neuroscience from bench to clinic.

## Background

Hearing loss affects approximately 1.5 billion people globally and imposes a significant burden on individuals and society, affecting communication, quality of life, and productivity [1]. Hearing loss may arise from developmental disorders, age-related changes and acquired pathological changes to the outer and middle ears (conductive hearing loss) or the cochlea and auditory nerve of the inner ear (SNHL). SNHL accounts for majority of all hearing loss [2]. Risk factors for SNHL include aging, excessive noise exposure, exposure to ototoxic or neurotoxic drugs, certain genetic variations, and other environmental causes [1]. Extensive research effort in past decades using small animal species (mice, guinea pig, gerbil) have led to our current understanding of the underlying mechanisms behind how these environmental and genetic risk factors can lead to SNHL. Despite this need, there is no effective pharmacological treatment available to reverse the pathology of SNHL, and to prevent or delay the progression of SNHL. Part of this has been attributed to difficulty of translating “proof-of-concept” for novel treatments established in small animal models to human therapies [3]. In this regard, we are one of several research groups that believe that such a translational gap may be closed more effectively by complementary uses of alternative, large animals (pig, sheep, and non-human primates) and started using them for auditory research in recent years. Among the choices of large animals, we and others have used sheep because of the availability and some advantages sheep have to offer. Here, we first provide a comprehensive comparison of data relating to the biology, auditory function, auditory system age equivalence, and ear anatomy in humans and the main laboratory species that have been used as animal models in auditory research. We then discuss the potential advantages and disadvantages of using sheep as a translational animal model in specific aspects of auditory research. Effective use of large animals in combination with small animals and in vitro model have a potential to accelerate the translation of the laboratory-based novel therapies for SNHL to the clinical setting.

## Main text

### Life span, body size and genetic background of experimental animals

The life span of laboratory animals is relatively short compared to humans (Table 1). The lifespan of small rodents, guinea pigs, and rabbits ranges from 2 to 8 years. Larger laboratory animals (sheep, pig, cat, dog, and monkey) have relatively longer lifespans ranging between 10 and 20 years (Table 1), with longer gestation periods ranging between 62 days in dog to 176 days in monkey (*Macaca fuscata*) compared to small laboratory animals. Monkeys (*Macaca fuscata,* mostly uniparous) and sheep (litter size ranging from 1.1 to 3.6, depending on the breeds) have smaller litter size compared to rodents [4, 5]. Small animals with shorter lifespan and larger litter size (ranging from 1 to 16 with average of 8.5 pups in mice and ranging from 1 to 13 with average of 9.1 pups in rats) [6, 7] make rodents suitable for expanding colonies when creating transgenic animals.

**Table 1:** Comparison of the life span and body weight in humans and animals

Species	Life span (years)	Birth weight (g)	Adult body weight	References
Human	73^a^	1972–3800^d^	62 kg^a^	Walpole et al. [8]
				Schild et al. [9]
				United Nations [10]
Sheep	14	2400–4000^c^	65–70 kg	Coop [11]
				Simmons et al. [12]
				Vicente-Pérez et al. [13]
Pig (Göttingen minipig)	15–20	350–570^c^	53 kg^a^	Köhn et al. [14]
				Ellegaard Göttingen Minipigs [15]
Dog	12–16^b^	231–270^c^	9–10 kg^c^	Heird et al. [16]
				Albert et al. [17]
				Choi et al. [18]
Monkey (*Macaca fuscata*)	10–11^c^	539–547^c^	8–11 kg^c^	Fooden and Aimi [19]
				Pflüger et al. [20]
Cat	11–18	65–165	3–6 kg	Gatel et al. [21]
				Kienzle and Moik [22]
				Teng et al. [23]
Rabbit	5–8	30–80	2–6 kg	Pritchett-Corning et al. [24]
				Sengupta and Dutta [25]
Guinea pig	4–7	70–90	500–800 g	Altman and Dittmer [26]
				Pritchett-Corning et al. [24]
Chinchilla	10–18	42–57	369–493 g	Spotorno et al. [27]
				Dzierzanowska-Goryn et al. [28]
				Bays [29]
Rat	2.5–3.5	5.7–7.3^d^	200–500 g^c^	Vehaskari et al. [30]
				Pritchett-Corning et al. [24]
				Sengupta [31]
Mice	1–3	1.82–1.86^e^	25–40 g	Pritchett-Corning et al. [24]
				Beauchamp et al. [32]

^a^Average value

^b^The data was adapted from the median survival and 10% survival age

^c^The range was adapted by merging the data from the male and female animals

^d^The range was obtained by mean ± 1 standard deviation

^e^The range was obtained by mean ± 1 standard error and the data was withdrawn from the figure in the reference

There are various inbred and outbred strains available in rodents, and most of their genome are comprehensively researched. Inbred strains of rodents can provide better stability of genotype and minimal individual difference. Some of the hearing loss related gene locus variation, such as *Ahl2, mt-Tr, and Cdh23*^*ahl*^ have been identified in some specific strains of mice [33]. The genetic background of most large experimental animals has not been fully researched. Over 850 breeds of sheep have been recognized worldwide [34]. However, the usage of different sheep breeds in biomedical research is reliant on their availability which can be regional. A survey on sheep usage in biomedical research showed that researchers tend to choose a sheep breed that is locally available, and more than half of the laboratory sheep user (51.2%) did not have a preference for a particular sheep breed [35]. The commonly used sheep breeds in biomedical research includes New Zealand Romney, Merino, Rambouilette, and Borderdale. To the best of our knowledge, there is no hearing loss related gene research has been done in sheep. Sheep genome have been sequenced [36], enabling genome editing technology to be used to generate genetic model for sheep [37]. We expect more information to become available about genomic data from different sheep breeds.

The adult body weight is an important factor when designing a suitable animal experiment with appropriate housing and space requirements, adjusting the amount of therapeutic compounds for systemic administration, and volume of tissues or blood that can be sampled. Human average adult body weight is approximately 62 kg [8] in the similar range to large animals like sheep (65–70 kg) and miniature pigs (53 kg) [8, 11, 14]. Other laboratory animals have much lighter normal adult body weight, ranging from 25 g in mice to 16 kg in dogs [18, 24] (Table 1). When it comes to birth weight, human babies are born with much higher body weight than most of the multiparous laboratory animals’, even if they have similar adult body weight, such as miniature pigs (Table 1). One animal that has similar birth weight as human is sheep, which are primarily uniparous like humans with occasional twins, with birth weight (2.4–4.0 kg) comparable to human singleton’s (2.0–3.8 kg) [9, 13] (Table 1). This similarity makes the sheep foetus a good animal model for various forms of paediatric research such as foetal pulmonary arterial hypertension [38] and foetal cardiac function assessment [39].

### Auditory thresholds and frequency range of laboratory animals and humans

**Fig. 1:**
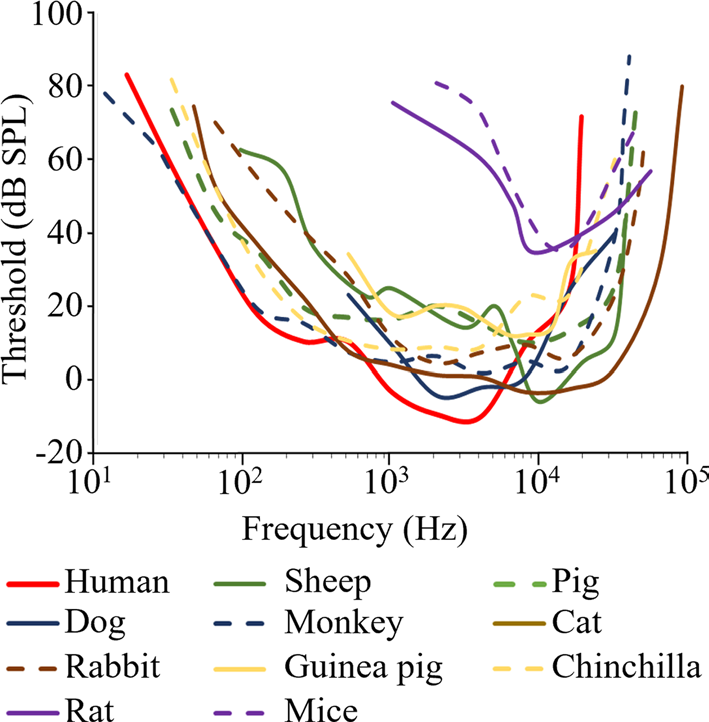
Comparison of audiogram in humans and animals. Plotted based on the reported audiogram of humans [40], sheep [222], pigs [44], dogs [49], monkeys (*Macaca fuscata*) [40], cats [52], rabbits [50], guinea pigs [51], chinchillas [45], rats [46] and mice (CBA/J) [53]. The audiogram of sheep, pigs, monkeys, cats, rabbits, and chinchillas were measured by behavioural testing. The audiogram of dogs, guinea pigs, rats, and mice were measured by electrophysiological testing. SPL: sound pressure level

Hearing in animals can be assessed using a variety of behavioural tasks or physiological measures for comparison with human performance. The estimation of auditory thresholds using the Auditory Brain Response (ABR) to tone bursts across frequency and Otoacoustic Emissions (OAE) are the most commonly used physiological assessment and which can be compared to human pure tone audiometry, or similar physiological measures. Here the hearing frequency range and sensitivity developed by pure tone audiometry in humans and ABR or behavioural testing in animals is compared (Fig. 1, Table 2). The frequency range of human hearing is from approximately 30 Hz to 18 kHz with maximum sensitivity around 2–4 kHz [40]. Clinically, the frequency-dependent variation in sensitivity is accounted for by the use of the dBHL scale, where 0 dBHL is the reference level of the mean normal hearing threshold at each frequency [41]. An auditory threshold of greater than 15 dBHL in humans is regarded as a clinical hearing loss [42, 43]. Laboratory animals used in hearing research have considerable variation in hearing range, and frequencies of maximum sensitivity (Fig. 1; Table 2). Sheep (100 Hz–30 kHz), monkey (*Macaca fuscata*, 28 Hz–37 kHz), and chinchilla (50 Hz–33 kHz) have a similar hearing range to humans [40, 44, 45]. Some small laboratory animals, such as rats (4–56 kHz), guinea pigs (86 Hz–47 kHz) and mice (2–88 kHz) can hear much higher frequency (higher than 30 kHz) sound and may have limited sensitivity to low frequency sound (lower than 500 Hz) compared to humans [46–48]. The frequency range with the maximum sensitivity to sound varies across species too; for example, humans have their greatest hearing sensitivity between 2 and 4 kHz with − 10 dB sound pressure level (SPL) of hearing threshold [40]. Monkey (*Macaca fuscata*, 1 dB SPL at 4 kHz), dog (0 dB SPL at 2–8 kHz), rabbit (4 dB SPL at 2 kHz), and chinchilla (8 dB SPL at 500 Hz–4 kHz) have their greatest sensitivity in a similar frequency range to humans [40, 45, 49, 50]. Other species, such as sheep (− 6 dB SPL at 10 kHz), pigs (9 dB SPL at 8 kHz), guinea pigs (12 dB SPL at 6–8 kHz), rats (35 dB SPL at 9 kHz), and mice (7 dB SPL at 16 kHz), have their greatest sensitivity at a higher frequency range [44, 46, 48, 51] (Table 2 and Fig. 1).

**Table 2:** Comparison of the hearing range and hearing threshold in humans and animals

Species	Hearing range (at 60 dB SPL)	Greatest hearing sensitivity frequency range^a^	Reference
Human	31 Hz–8 kHz^c^	− 10 dB SPL at 2–4 kHz^b,c^	Jackson et al. [40]
Sheep	100 Hz–30 kHz^c^	− 6 dB SPL at 10 kHz^b,c^	Wollack [222]
Pig	42 Hz–41 kHz^c^	9 dB SPL at 8 kHz^b,^^c^	Heffner and Heffner [44]
Dog	67 Hz–45 kHz^c^	0 dB SPL at 2–8 kHz^b, d^	Heffner [54]
			Poncelet et al. [49]
Monkey (*Macaca fuscata*)	28 Hz–37 kHz^c^	1 dB SPL at 4 kHz^c^	Jackson et al. [40]
Cat	58 Hz – 75 kHz^b,c^	− 3 dB SPL at 8–16 kHz^b, c^	Heffner and Heffner [52]
Rabbit	360 Hz–42 kHz^c^	4 dB SPL at 2 kHz^b,c^	Heffner and Masterton [50]
Guinea pig	86 Hz–47 kHz^c^	12 dB SPL at 6–8 kHz^b,d^	Heffner et al. [47]
			Naert et al. [51]
Chinchilla	50 Hz–33 kHz^c^	8 dB SPL at 500 Hz–4 kHz^b,c^	Heffner and Heffner [45]
Rat	4–56 kHz^d^	35 dB SPL at 9 kHz^b,d^	Syka [46]
Mice	2–88 kHz^c^	7 dB SPL at 16 kHz^c^	Heffner et al. [48]

SPL: sound pressure level

^a^Defined by the trough of the audiogram

^b^Data was withdrawn from the audiogram in the reference

^c^Determined by behavioural testing

^d^Determined by electrophysiological testing

**Fig. 2:**
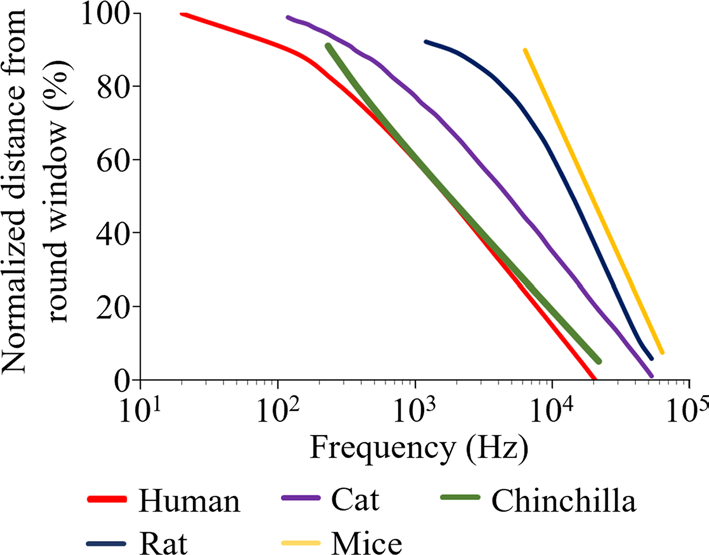
Comparison of tonotopic map of cochlea in humans and animals. Plotted based on the reported tonotopic map of cochlea in humans [56], cats [57], chinchillas [58], rats [59], and mice [53]. The ordinate is normalized by Min–Max scaling. The distance from round window to the apex of the cochlear turn along the basilar membrane length is normalized to 1 (100%)

Frequency representation in the cochlea is organised spatially by the tonotopic map along the length of the cochlea such that higher frequencies are detected at the basal end, while more lower frequency sounds are detected towards the apical tip of the cochlea [55]. In humans, 30% from the apex (low frequency region) and 30% from the basal (high frequency region) extremes, correspond to approximately 20–500 Hz and 4–20 kHz, respectively, while equivalent regions in mice correspond to approximately 6–10 kHz and 32–64 kHz, respectively [53, 56] (Fig. 2).

The difference in hearing range and tonotopic representation within the cochlea may differentially influence animal’s response to the same environmental noise and development of noise-induced hearing loss models. In human occupational noise-induced hearing loss, the main auditory threshold shift is at 3–6 kHz (corresponding to the basilar membrane at approximately 25–40% from the basal of cochlea) with progression to predominately higher frequencies followed by lower frequencies [56, 60]. However, in rats, long-term, low-level noise exposure (90 days of 90 dBlin 4–20 kHz wide band noise for 4 h/day, 5 days/week) caused the hearing threshold shift primarily in the 12.8–16.3 kHz frequency range (corresponding to the basilar membrane at approximately 45–50% from the basal of cochlea) and no significant threshold shift was noticed at the 4–8 kHz frequency range [59, 61] Therefore caution is required in interpreting the noise-induced SNHL in rodents if trying to place this condition in a context of environmental or occupational noise exposure in humans. In addition to tonotopic map, ear canal resonance may also affect the outcome of the hearing threshold shift after noise exposure in different species. For example, in humans, rats, and mice, the ear canal resonance enhances the sound pressure at 2–5, 19.1, 20–25 kHz, respectively [62–64]. Therefore, even exposed to similar noise, rodents with ear canal resonance at higher frequency may led to the hearing threshold shift occurs at different frequency range compared to humans.

In other types of SNHL independent of noise, the difference in the frequency range may matter less. For example, in humans, cisplatin-induced SNHL affects the hearing threshold at in high frequencies (> 4 kHz) the most with hair cell loss observed in high frequency region of the cochlea [65, 66]. In mice, cisplatin similarly increased hearing thresholds at the high frequencies (32–40 kHz) with hair cell loss at the high frequency region of the cochlea and similar histopathologic changes [67]. There have been only a few studies where large animals were used for modeling acute noise-induced hearing loss. Sai et al. [68] exposed 2–3 months-old miniature pigs to white noise at 120 dB for 3 h on 2 consecutive days. The increased the ABR threshold shift at 4–8 kHz was more severe than other tested frequencies (2–24 kHz). Gerhardt et al. [69] investigated the effect of noise on foetal sheep, exposing pregnant ewes to 120 dB SPL noise for 16 h per day for 1 day or 4 consecutive days. This resulted in increased ABR threshold to click and tone burst stimuli (0.5 kHz, 1 kHz, and 2 kHz) and hair cell loss occurred in the middle to apical turn of the cochlea in fetal sheep. To the best of our knowledge, the effect of long-term low-level noise exposure in large animals has not been established yet.

### The auditory system at equivalent age

When using animal models for human medical research, it is important to correlate approximate equivalent age and stage of development between species [70]. Several different approaches to compare the age-equivalence between humans and rodents have been published [71, 72]. In these approaches, various parameters were used for correlating the age equivalence, such as lifespan, molar ageing method, and weight of eye lens. However, the age equivalence differs among the different approaches. Therefore, it is hard to establish the auditory system age equivalence without an approach that includes the comparison of the auditory system developmental and aging milestones between humans and animals. Here we summarized some of these milestones in humans, sheep, rats, and mice from available studies (Table 3) to help correlate age equivalence for the auditory system. In humans, the differentiation of sensory hair cells, critical sensory component of the cochlea, occurs at their 26% of gestation period [73]. In rats [74] and mice [75], the hair cell differentiation occurs at much later gestation periods than humans (91% and 78%, of gestation period respectively). In humans [76] and sheep [77], ABR potentials can be recorded at 63% and 72% of their gestation period, respectively. In contrast, the ABR potential in rats and mice can only be detected after birth (Table 3). The ABR wave latency matures at 16 months, 49 days, 56 days, and 30 days after birth in humans, sheep, rats, and mice, respectively [78–81]. In humans, children show adult-like ABR threshold at 2 years of age [82]. Unlike humans, the ABR threshold reaches the adult-like pattern in rats and mice before the ABR latency maturation (Table 3).

**Table 3:** Auditory system age equivalence in humans and animals

Events/species	Cochlear hair cell differentiation	ABR onset^a^	ABR latency maturation^b^	ABR threshold maturation^c^	ARHL onset	Reference
Human	GD 74, 26% of gestation	GD 175, 63% of gestation	16 M	2 Y	60–69 Y^d^	Hecox and Galambos [78]
						Starr et al. [76]
						Sasama [82]
						Pujol et al. [73]
						Bainbridge and Wallhagen [83]
Sheep	NA	GD 106, 72% of gestation	49 D	NA	NA	Wolfson et al. [77]
						Griffiths et al. [81]
Rat	GD 20, 91% of gestation	12.5 D	56 D	22 D	12—over 30 M^e^	Church et al. [79]
						Geal-Dor et al. [84]
						Zine and Romand [74]
						Popelar et al. [85]
						Qiu et al. [86]
Mice	GD 15.5, 78% of gestation	12 D	30 D	14 D	3—over 24 M^e^	Song et al. [80]
						Sha et al. [87]
						Chonko et al. [75]
						Keithley et al. [88]

ABR: Auditory brainstem response; ARHL: age-related hearing loss; D: days after birth; GD: gestational days; M: months after birth; NA: not available; Y: years after birth

^a^Defined by the earliest time point that the ABR potential can be recorded

^b^Defined by the earliest time point that the adult-like ABR latency can be recorded

^c^Defined by the earliest time point that the adult-like ABR threshold pattern can be recorded

^d^Defined by the prevalence of AHRL excess 30% in the studied population

^e^Depending on the strains

Age-related hearing loss (ARHL) or presbycusis is observed in humans, small rodents, and non-human primates. In humans, the prevalence of ARHL increases non-linearly with age and exceeds 30% within the 60–69 year old populations [83]. In rhesus monkeys, the average ABR pure tone threshold increases at the age of 15 and keeps increasing with age [89]. The ARHL onset for rodent is more complicated as it differs significantly between strains with different genetic backgrounds in rats [85] and mice [90]. For example, in mice, at least 10 inbred mice strains share the *Ahl* variation which is responsible for the early-onset ARHL occurring at 3 month after birth [91, 92]. In contrast, the ARHL onset in ARHL-resistance mice strains, such as CBA/CaJ strain, can only be detected after 15 months of age [93]. The ARHL onset difference caused by gene variants makes the age-equivalence between the ARHL rodent model and human ARHL hard to be defined. Unfortunately, except for some non-human primate species [89, 94], there is little data currently available to define the age-related decline in auditory function in large animal models (such as sheep and pig). Such data would be useful to fully take advantage of the longevity of large animals in the context of ARHL.

### Comparison of inner ear anatomy in humans and laboratory mammals

**Fig. 3:**
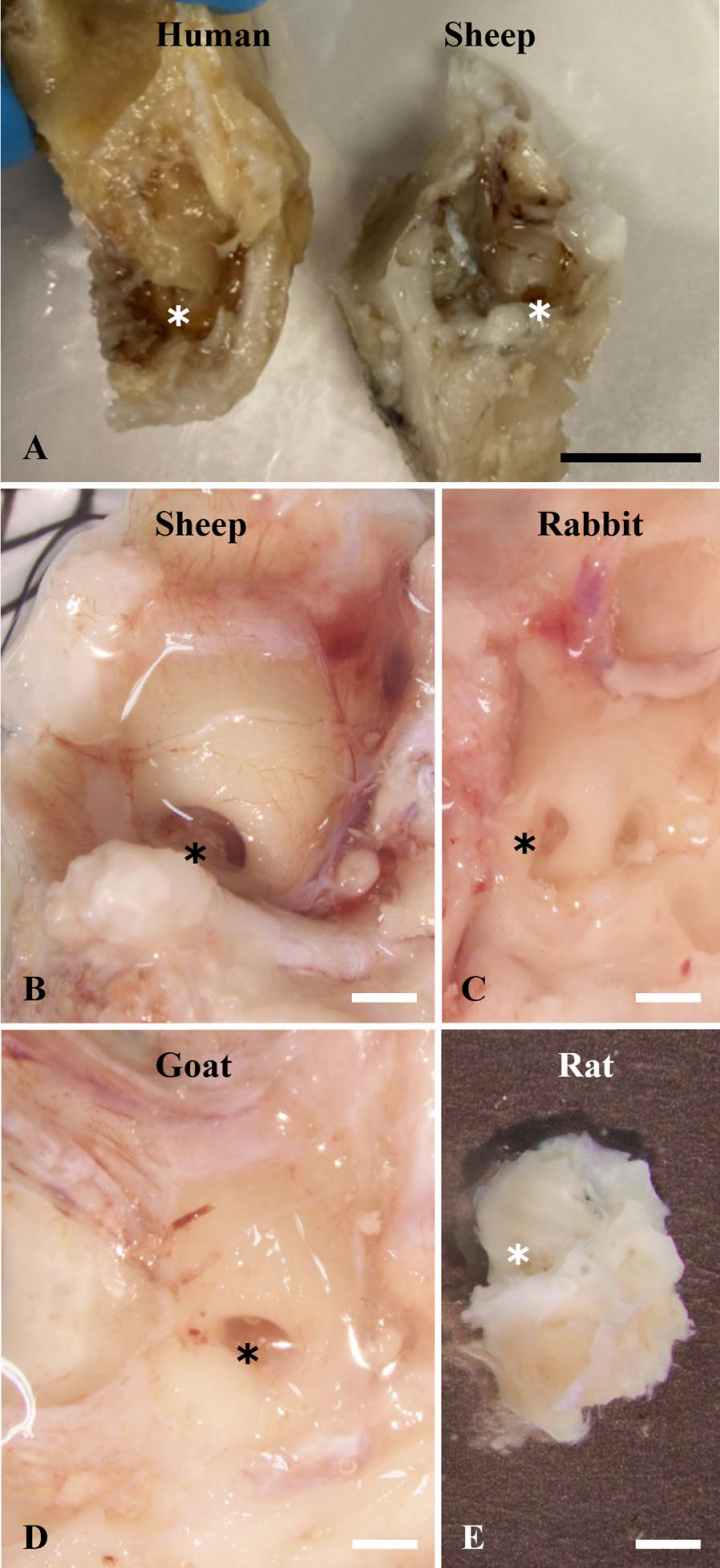
Comparison of cochlea in humans and animals. Sheep (right cochlea) share similar cochlear size with humans (left cochlea) (**A**). Sheep (**B**), rabbit (**C**), goat (**D**), and rat (**E**) cochlea are demonstrated on the same magnification for comparison. Black scale bar: 1 cm; asterisk: round window; white scale bar: 500 μm

Here we will compare the main laboratory animal species and humans, focusing on the geometry and some of the anatomical features of the cochlea. The cochlea of humans is approximately 6 mm in diameter and is embedded in the temporal bone. It is a fluid-filled structure and consists of 3 fluid filled compartments (scala vestibuli, scala media, and scala tympani). The cochlea of mammals all shares the same basic spiral structure, however the length of the cochlea and the number of turns differs considerably. The human cochlea spirals 2.6 turns around the modiolus [95], whereas guinea pig, miniature pig, and dog have more cochlear turns (3.5–4.25 turns) and rats and mice have fewer cochlear turns (1.75–2.2 turns) compared to humans [96–99]. The cochlear length in dog, monkey (*Common marmoset*), cat, rabbit, guinea pig, chinchilla, rats, and mice (5–24 mm) are shorter compared to human (32 mm) [53, 98–103]. Also, the scala tympani (ST) volume in most of the animal species, such as guinea pig (4.76 µl), monkey (*Common marmoset*, 5.22 µl), mice (0.32 µl), and rats (1.04 µl) are substantially smaller compared to humans (29.22 µl) [102, 104]. In contrast, the sheep has very similar cochlear structures compared to humans, with similarities in the number of cochlear turns (2.25 turns), cochlear length (34.1 mm), and ST volume (25.04 µl) [105] (Table 4 and Fig. 3).

**Table 4:** Comparison of the cochlear turns, cochlear length, and scala tympani volume in humans and animals

Species	Cochlear turns	Cochlear length (mm)	Scala tympani volume (µl)	Reference
Human	2.6	32^a^	29.22	Keen [98]
				Thorne et al. [104]
				Erixon et al. [95]
				Kuthubutheen et al. [106]
Sheep	2.25	34^a^	25.04	Schnabl et al. [105]
Miniature pig	3.5	39^b^	NA	Yi et al. [97]
Dog	3.5	24^a^	NA	Keen [98]
				Le and Keithley [100]
Monkey (*Common marmoset*)	2.84	17^b^	5.22	Johnson et al. [102]
Cat	3	20^a^	NA	Keen [98]
				West [96]
Rabbit	2.25	15^a^	NA	Axelsson and Lind [103]
				Yuan et al. [107]
Guinea pig	4.25	21^a^	4.66	Keen [98]
				West [96]
				Thorne et al. [104]
Chinchilla	3	18^a^	NA	West [96]
				Bohne and Carr [101]
Rat	2.2	9^a^	1.04	Burda et al. [99], Thorne et al. [104]
				Yi et al. [97]
Mice	1.75	5^a^	0.32	Burda et al. [99]
				Thorne et al. [104]
				Müller et al. [53]

NA: Not available

^a^Measured along the basilar membrane

^b^Scala tympani length

The mammalian cochlea has two types of hair cells, inner hair cell (IHC) and outer hair cells (OHC) in the organ of Corti (OoC). The afferent innervation by the spiral ganglion neurons (SGNs) transmit sound from the hair cell to the brain [108]. In SNHL, the age-dependent decline in IHC and OHC number is well documented in humans, and the loss of OHC/IHC is the hallmark of sensorineural hearing loss [109]. Degeneration of SGN is usually preceded by IHC/OHC loss in both human and animal models [110]. In cochlear implant recipients, hair cell density and SGN survival are correlated with hearing outcomes [111]. The IHC and OHC are located on the basilar membrane in the OoC in a highly ordered manner and except in the apical region at every point along the OoC, there are one row of IHC and three rows of OHC. The IHC and OHC density are similar among most of the laboratory animal species and humans (Table 5). The IHC and OHC count in different animals generally correlates with the length of the cochlea with those animal species with a longer length of cochlear duct usually have higher numbers of IHC and OHC (Tables 4 and 5).

**Table 5:** Comparison of the inner hair cell, outer hair cell, and spiral ganglion neuron count/density in humans and animals

Species	Inner hair cell count^a^	Outer hair cell count^a^	Spiral ganglion neuron count^a^	References
	(Inner hair cell density^b^)	(Outer hair cell density^b^)	(Spiral ganglion neuron density^c^)	
Human	2936	11,650	33,383	Úlehlová et al. [112]
	(86)	(343)	(3.0–5.3)	Nelson and Hinojosa [113]
				Suzuki et al. [114]
Dog (beagle)	2775	10,702	NA	Sampaio et al. [115]
	(101)	(389)	(13.9–19.9)	Malkemper et al. [116]
Cat	2723	10,105	48,957^d^	Lee et al. [117]
	(99)	(373)	(NA)	Malkemper et al. [116]
Rabbit	1556	5,522	NA	Yuan et al. [107]
	(107)	(381)	(NA)	
Monkey (*Saimiri sciureus*)	2134	8056	NA	Dayal and Bhattacharyya [118]
	(NA)	(NA)	(NA)	
Guinea pig	2056	7377	NA	Burda [119]
	(100)	(361)	(17.9–18.4)^e^	Wrzeszcz et al. [120]
Chinchilla	1827	7400	NA	Bhattacharyya and Dayal [121]
	(100)^d^	(400)^d^	(12.1–13.2)	Bohne and Carr [101]
				Takeno et al. [122]
Rat	959	3740	16,450	Keithley and Feldman [123]
	(NA)	(NA)	(15.7–18.6)	Burda et al. [99]
				McGuinness and Shepherd [124]
Mice	726	2466	7380	Burda et al. [99]
	(121)	(411)	(35.5–39.4)^d^	Irving et al. [125]

NA: Not available

^a^Number of cells per cochlea, unless specified

^b^Cellular densities in number of cells/mm along the length of the basilar membrane or organ of corti

^c^Cellular densities in number of cells/0.01 mm^2^

^d^Data withdrawn from the table/figure in the reference

^e^Range adapted from mean ± 1 standard error

Spiral ganglion neurons are the primary afferent neurons and the SGN cell bodies are located in the modiolus, specifically Rosenthal’s canal, of the cochlea. Humans have a higher SGN count than rats and mice and a lower SGN density than dog, guinea pig, chinchilla, rats and mice (Table 5). The number of SGNs or densities of SGNs in large animals is not well characterized. SGNs numbers decrease gradually with age in human and in rodent models of ARHL [126].

The round window (RW) is one of two membrane-bound openings on the bone surrounding the cochlea, separating the middle and inner ears. The RW is covered by an epithelial membrane, round window membrane (RWM), and is commonly used as access to the cochlea from the middle ear in cochlear implantation surgery [127], inner ear drug delivery [128], and drug diffusion after intratympanic injection [129]. With intratympanic injection, compounds administered into the middle ear space enters the inner ear via the RWM predominantly by passive diffusion. Intratympanic injection is emerging as the method of choice for local delivery of therapeutic molecules to the inner ear in recent clinical trials [130]. In this context, the thickness and the surface area of RWM have been reported as the key factors affecting drug diffusion efficacy from the middle ear to the inner ear [131]. The round window membrane (RWM) is a triple-layered membranous structure at the end of the ST, separating the middle ear from the inner ear [132]. The RWM thickness in humans (69–70 µm) is thicker than that of most small animals such as mice (9–11 μm), rats (9–14 μm), chinchillas (11–17 µm), and guinea pigs (30–53 μm) [133–137]. Sheep (56–74 µm) and monkey (*Rhesus macaque,* 40–60 µm) have similar RWM thickness to humans [138, 139]. Also, the RWM surface area in humans (2.98 mm^2^) is larger than chinchilla (1.06 mm^2^) and guinea pig (1.18 mm^2^) and similar to sheep (approximately 3.79 mm^2^) and cats (approximately 1.77–3.14 mm^2^) [138–143] (Table 6). The difference of RWM thickness and the surface area between different animal models and humans are important factors to be considered when translating findings from studies of the intratympanic approach for local delivery of therapies in live animal models to humans.

**Table 6:** Comparison of the central RWM thickness, RWM diameter, and RWM surface area in humans and animals

Species	Central RWM thickness (µm)	RWM diameter (mm)	RWM surface area (mm^2^)	Reference
Human	69–70^a^	Long axis: 2.1	2.98 ± 0.43^b^	Sahni et al. [133]
		Short axis: 1.8		Takahashi et al. [140]
				Zhang and Gan [144]
Sheep	56–74^a^	Long axis: 2.3	3.79^c^	Han et al. [138]
		Short axis: 2.1		
Monkey (*Rhesus macaque*)	40–60	NA	NA	Goycoolea et al. [139]
Cat	20–40	1.5 – 2.0	1.77–3.14^d^	Goycoolea et al. [143]
Guinea pig	30–53	Long axis:1.3	1.18 ± 0.08^b^	Ghiz et al. [142]
		Short axis: 0.9		Gan et al. [137]
Chinchilla	11–17^a^	Long axis:1.4	1.06 ± 0.23^b^	Schachern et al. [136]
		Short axis: 1.0		Vrettakos et al. [141]
Rat	9–14^a^	NA	NA	Yoon and Hellstrom [135]
Mice	9–11^a^	NA	NA	Kitamura et al. [134]

RWM: Round window membrane; NA: Not available

^a^Range adapted from mean ± 1 standard deviation

^b^Data in mean ± standard deviation

^c^Calculated from the diameter data with assumption of the RWM is in shape of a ellipse

^d^Calculated from the diameter data with assumption of the RWM is in shape of a circle

### Utility of animal models in development of the cochlear implant

Research focussed on cochlear implants is emerging as an important area for use of large animal models and warrants consideration here. Recreating pathologies of certain types of SNHL in rodent and guinea pig models have served as a useful paradigm for understanding the mechanisms behind noise-induced [145], ototoxic [146], and aging-related [147] SNHL, as well as contribution of genetic variations to SNHL [148]. There are excellent reviews by Bowl and Dawson [147] and Escabi et al. [145] on the use of rodent models in understanding the pathophysiology of SNHL. Here, we focus on the use of different animal models for CI studies. The CI offers bionic hearing to individuals with moderate to severe sensorineural hearing loss by bypassing damaged hair cells in the cochlea and directly stimulating SGN. Rodents, guinea pigs and cats have been extensively used in CI developmental research, playing an important role in establishing the safety and efficacy of CI [125, 149]. In a historic context, the use of cats with congenital deafness as an animal model for human hearing impairment has provided a valuable pre-clinical evidence for safety and efficacy of CI in restoring hearing capabilities [150, 151]. Also, the efficacy of CI was further demonstrated in early-onset of ARHL of mice [152]. Pre-clinical evidence from these small animal models have led to human clinical trials and implementation of CI now as highly successful surgical treatment available to restore hearing in patient with deafness or profound hearing loss.

**Fig. 4:**
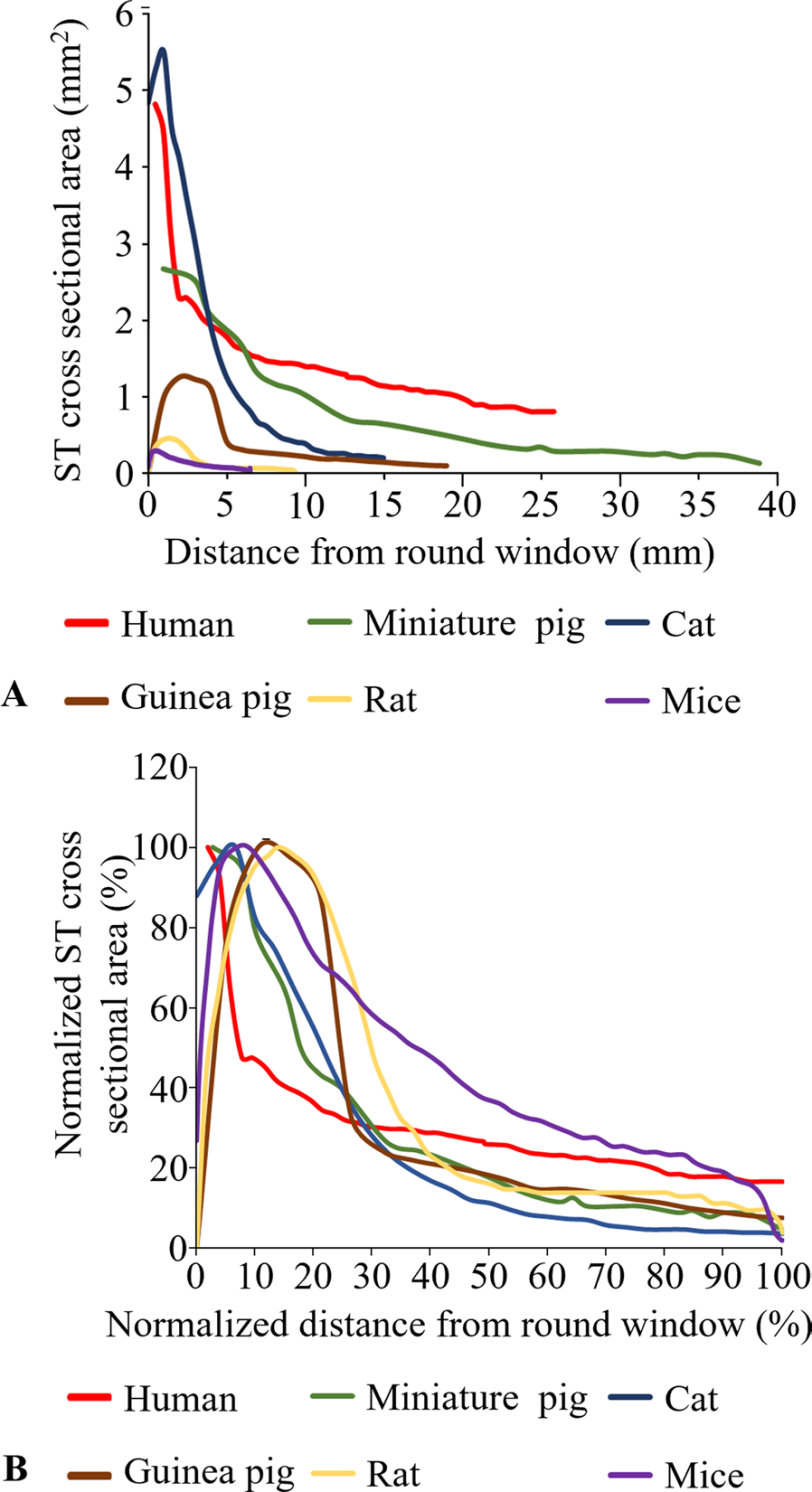
Comparison of scala tympani cross-sectional area in humans and animals. Plotted based on data from humans [167], miniature pigs [168], cats [167], guinea pigs [117], rats (Wistar), and mice (NMRI) [99]. The abscissa is the distance in millimetre from the round window along the cochlear basilar membrane (**A**). The ordinate and abscissa are normalized by Min–Max scaling to 1 (100%) (**B**). ST: scala tympani

Current ongoing effort in CI research is the development of new or improved devices to deliver better hearing outcomes in CI patients, improving neuronal (SGN) survival, as well as understanding post-CI pathophysiological responses in the cochlea such as inflammation and fibrosis, which is thought to negatively impact the CI outcomes. In this regard, reproducible surgical approaches for CI implantation have been well established in small (e.g., rodent and guinea pig) animal models [153, 154]. For example, small animal models are widely used to investigate the foreign body response after CI surgery [155]. However, some limitations for the use of small animals and cats due to the difference in cochlear size and genetic variability have been pointed out [156, 157], and alternative large animals may be useful addition to the research effort. Large animal models such as sheep [157] and minipig [156, 158] are emerging for translational CI research. Table 7 summarises how different size cochlea in animal models compared to human influences the number of CI electrodes that can be tested. The commercial CI electrode used in humans contains 15–22 active contacts [159]. The length of the CI electrode array varies from 20 to 31 mm, yielding a mean insertion depth angle from 341 ± 22 to 673 ± 38 degrees [160]. In rodents, the inserted CI length (< 6 mm), angle (~ 270° in guinea pig), and the number of inserted electrodes (< 6 electrodes) are less than in humans (Table 7). This difference of the CI models used in small animals and humans can be explained by the difference of the ST cross-sectional area and the cochlear length in each species. The ST cross-sectional area decreases to less than 1 mm^2^ within 5 mm in depth along the cochlear length in most small laboratory animals. In humans and miniature pigs, the ST cross-sectional area stays larger than 1 mm^2^ at 10 mm and 25 mm in depth along the cochlear length, respectively (Fig. 4A). Therefore, the full-sized human CI is generally too large in cross-sectional area and too long to be used in small rodents. In the normalized ST cross-sectional area plot (Fig. 4B), humans’ ST cross-sectional area decreases rapidly within the first 10% of the total cochlear length from the basal turn and stays in 20–40% of the maximum ST cross-sectional area along the rest of the cochlear length. The ST cross-sectional area in laboratory animal species increases to their maximum value in the first 10–20% of the cochlear length from the basal turn and decreases to less than 10% of the maximum the cross-sectional area in a relative slow slope compared to humans’ all the way to the apex end of the cochlea. Therefore, in humans, the CIs with a cross-sectional area smaller than 20% of the maximum value of the ST cross-sectional area can potentially be inserted into the apex end of the cochlea. In most laboratory animals, except for mice, the CIs with cross-sectional area of their 20% of the maximum ST cross-sectional area can only be inserted to 40–60% of the cochlear length. On the other hand, large animals with larger ST volume (Table 4) potentially have an advantage as commercial and full-sized human CIs can be used without adjustment. In study by Kaufmann et al. [157], the full size CIs were inserted into the cochleae of adult female sheep for 30 days. Only limited post operative complications were noticed and the ABR and round window electrocochleography were recorded successfully in those sheep. Also, in studies by Schnabl et al. [105], full size human CIs could be adapted for the use in sheep and the insertion depth (7–18 mm), angle (540°), and the number of inserted electrodes (16 electrodes) were similar to the human CI surgery values. It should be noted that in the study by Kaufmann et al. [157], only partial (4.6–12 mm) CI insertion could be achieved in sheep due to the narrowing of scala tympani in the second turn of the cochlea. It is also noteworthy that minipig can also be used in similar way as the CI model [156, 158].

**Table 7:** Comparison of the cochlear implantation used in human and laboratory animal studies

Species	Cochlear implantation model	Inserted length (mm)	Insertion depth angle (°)	Number of inserted electrodes	Electrode diameter (mm)	References
Human	FLEX^20^, FLEX^24^, FLEX^28^, and Standard electrode arrays, Med-El	20–31	341–673	NA	NA	Franke-Trieger et al. [160]
Human	FLEX^28^ array, Med-El	NA	525	11	0.8	Kuthubutheen et al. [106]
Human	FLEX^31^ array, Med-El	NA	489	11	0.5–1.3^a^	Kuthubutheen et al. [106]
Human	Contour Advance, Cochlear Limited	17	NA	NA	0.5–0.8^a^	Nguyen et al. [161]
Human	Hybrid-L, Nucleus®	16	NA	NA	0.25–0.55^a^	Nguyen et al. [161]
Human	Flex EAS, Med-EI	18	NA	NA	0.35–0.8^a^	Nguyen et al. [161]
Sheep	Cochlear slim straight 522 full implant, Nucleus®	7	540	NA	0.6	Kaufmann et al. [157]
	Flex24 demonstration electrode array, Med-El					
	EVO demonstration electrode array, Oticon medical					
Sheep	FlexEAS and Standard electrode array, Med-El	18	NA	16	0.8	Schnabl et al. [105]
Monkey (*Macaca Mulatta*)	Medium electrode array, Med-El	9–27	190–720	6–12	0.5–0.8^a^	Marx et al. [162]
Monkey (*Common marmoset*)	H12, Cochlear Limited	8	270	10	0.4 × 0.25^b^	Johnson et al. [102]
Cat	CI24 cochlear implant, Nucleus®	8	180	8	0.7	Fallon et al. [163]
Cat	Hybrid-L24 electrode array, Cochlear Limited	11	335	16	0.25–0.5^a^	Shepherd et al. [164]
Cat	CI24 cochlear implant, Nucleus®	6	176	8	0.37–0.5^a^	Shepherd et al. [164]
Guinea pig	NA	4	NA	2	0.3–0.5^a^	Honeder et al. [153]
Guinea pig	Custom-made research CI electrodes, Med-El	5	270	6	0.5	Andrade et al. [165]
Guinea pig	Custom-made research CI electrodes, Med-El	4	270	5	0.5	Andrade et al. [165]
Mice (C57BL/6)	NA	2	NA	4	0.21–0.27^a^	Navntoft et al. [154]
Mice (C57BL/6)	Cochlear HL03, Cochlear Limited	2	NA	3	0.15	Claussen et al. [166]
Mice (C57BL/6)	NA	2	NA	3	0.2–0.8	Irving et al. [125]
Aachen minipigs	Flex ^20^, Med-El	20	NA	19	0.3 × 0.5 ^b^–0.8^a^	Yildiz et al. [156]

NA: Not available

^a^Data expressed in the diameter range from the tip to the base of the inserted electrode

^b^Data expressed in the width × high of electrode

The size and geometry of the cochlea is critical for fitting human CI. If the study needs to take into account of the feasibility of CI surgery procedure, then the overall anatomy of the skull and temporal bone becomes a relevant factor to be considered for selecting the right large animal model. While the cochlear length in pig is long enough for a full length human CI, the temporal bone anatomy of pig head is not ideal for CI surgery [97]. The length and location of external auditory canal is significantly different to those of human, and the mastoid is positioned partially beneath the atlanto-occipital joint in pig [169]. In the study by Schnabl et al. [105], the author compared the feasibility of using sheep and pig as the CI animal model. A thick layer of mixture of soft tissue and fatty tissue covered on the pig mastoid made the CI surgery approach difficult. In contrast, sheep was a better animal for CI surgery because of its similarity to human inner and middle structure and only a thinner layer of tissue covered on mastoid. In addition to ongoing research on sheep CI models, new technologies like vestibular implant [170] and brainstem implants [171, 172] are also underway. Large animal models, such as the sheep and minipig model, may offer a translatable platform for development of these novel implantable devices.

### Advantage and disadvantages of sheep and other large animal model in auditory research

We have summarised data available to allow comparison of laboratory animal models for purpose of identifying advantages and disadvantages that large animal models may offer over the conventional small animal models used in auditory research. These advantages and disadvantages are summarized below.

#### Advantages

The first potential advantage is the longevity of larger animals used in research. An example of the importance of this advantage comes from models of human neurodegenerative disease. Age-dependent amyloid β deposits and *tau* neurofibrillary accumulations are the hallmark signatures of Alzheimer’s disease (AD) [173]. This signature of AD does not naturally occur in aged wild type mice [174]. Therefore the rodent model for AD require a transgenic animal that overexpresses human amyloid precursor protein to mimic AD pathology in mice [175]. In contrast, when sheep are allowed to age on the farm, the brain (extracted from 8 to 12 year-old sheep) has comparable amyloid and neurofilament accumulation and progressive appearance of amyloid plaques as observed in humans with AD [176–179]. The distribution of *tau* tangles in sheep is mostly in the temporal lobe and entorhinal cortex [178], which is similar to the typical AD *tau* pattern in humans [180–183].

Aging is a major contributor to hearing loss in humans [1], and ARHL progresses slowly over many decades at an approximate rate of 1 dBHL (decibel hearing level)/year above the age of 60 years in human [184, 185]. In human ARHL, the IHC and OHC loss progresses from the high frequency region of the cochlea towards the low frequency region [186]. This hair cell loss pattern also corelates with the progressive pattern of changes in pure tone hearing thresholds with ARHL, which begins at the 6–8 kHz and progresses towards lower frequencies (2–4 kHz) with age [187]. While this functional decline of the auditory system can be observed in a compressed period of time in rodents [188, 189], how well it reflects on human ARHL remains as a question. In aging Fisher Brown Norway rats, the OHC loss begins from the low and middle frequency regions of the cochleae. The IHC count is intact until 28 months of age. However, the ABR threshold increases evenly at all tested frequencies (2–24 kHz) with age [190]. In aging CBA/J mice, the OHC loss starts from the low frequency regions of the cochleae, followed by the middle and high frequency regions during their mid to late lifespan (~ 25 months of age). The IHC count change is insignificant until they are 25 months of age. The corresponding change in ABR thresholds begins at 4 kHz (apex turn) at 3 months of age and is followed by changes at 12 kHz and 24 kHz (apex to middle turn) at around 12 months of age and 48 kHz (basal turn) at 24 months of age [87]. The pattern of ARHL, hair cell loss, and SGN loss in larger animal models has not been extensively characterized to date, and as such the characteristics of aging-related pathologies in the inner ear of large animals remain unclear. Once these data become available, large animals such as sheep may become a suitable model for investigations of the age-dependent pathology of presbycusis, and to study the interaction between SNHL with other age-related chronic morbidities, such as Alzheimer’s disease (AD) where hearing loss in mid-life is regarded as a significant risk factor in humans [191].

In addition, the longer gestation period of large animals may also be an advantage in studying congenital/early onset hearing loss. Some prenatal risk factors such as asphyxia, maternal infection, ototoxic drug exposure, and low for gestational birth weight [192–194] cause hearing impairments in human neonates. Sheep foetuses at 2–3-month-old [195] have similar overall middle ear ossicle size and shape to human neonates [196] which carries onto adulthood [105, 197]. The similarity of the development and maturation of the auditory system between sheep and humans makes sheep a potential animal model option for researching the pathogenesis behind the prenatal hearing loss risk factors and other obstetric or paediatric related hearing impairment. For example, Smit et al. [198] used pregnant sheep to confirm the hypothesis that in utero inflammation causes a perilymphatic inflammatory response and impaired hearing in the fetus. Griffiths et al. [199] utilized a sheep experimental paradigm to illustrate that external sounds can penetrate the uterus and result in alterations of the foetal auditory brainstem response.

Second advantage that large animals have to offer is the size and the anatomical similarities of the peripheral auditory system between large animals and human as we have summarised in this review. Our comparison shows that in terms of the cochlear size anatomical features (such as RWM), and fluid volume inside the cochlea, large animals are more similar to human than small animals. These features make large animals more translatable models for development of devices or procedures for the inner ear. As mentioned, sheep animal model has an advantage as commercial and full-sized human CIs can be used without adjustment [105]. More recently, sheep has been used to demonstrate feasibility of ultrasound probe inserted through the middle ear cavity [200]. In addition to devices and procedures, the volume of inner ear fluid is important when designing or comparing studies that utilises perilymph sampling for the pharmacokinetic analysis or biomarker discovery, or for drug delivery to the inner ear. Due to the connection between the inner ear fluid and the cerebrospinal fluid (CSF) via the cochlear aqueduct, contamination of the inner ear fluid samples with CSF is one of the main obstacles [201]. The CSF contamination is more prominent in small laboratory animals, since they have relatively small volume of perilymph in ST than humans and larger laboratory animals [202]. The animal species with relative similar body and organ size and blood volumes with humans may have advantage for pharmacokinetic studies and blood-born/tissue-based biomarkers [203, 204].

#### Disadvantages

On the flip side to the advantage of the longevity, the longer gestation period and the smaller litter size can be the major disadvantage of large animals such as sheep and monkey, if research requires development of transgenic animal models to recreate certain SNHL pathologies and higher throughput research. Rodents have a shorter gestational period (22–23 days) and larger litter size (7–12) [205]. Genetically modified animal models are much easier and cheaper to be generated in rodents, and have been extensively used in auditory research; Slc26a4 gene mutation to estimate the feasibility of gene therapy [206], Rfx1/3 conditional knock-out (cKO) to illustrate the essential role for regulatory factor X in hearing [207], just to name a few examples. Although transgenic large animal models are limited, recent advances in CRISPR/Cas9 gene editing technologies made generation of transgenic large animals more feasible; there are now transgenic models of sheep and goat [208–210] and primate [211, 212]. CRISPR-modification of otoferlin gene, critical for hearing function, in sheep has also been demonstrated recently [37]. It is also important to note that in mice, it is well known that different strains of rodents exhibit different auditory function and hearing loss [213–215]. For example, both C57BL/6 [216, 217] and DBA/2J [218] strain mice have Cadherin 23 abnormalities which cause early-onset ARHL. CAST/Ei strain mice is another example, homozygous for the resistance allele (+ *ahl*/+ *ahl*) that confers resistance to hearing loss compared to CBA/CaJ strain mice, and have no detectable hearing loss even after their 24 months of ages [88, 214]. While mouse strains are extensively characterized, similar strain variability and genetic background information is yet to be scrutinized for large animals. As such, the strain differences in genetic background and its impact on hearing function need to be considered when interpreting data arising from any animal models but particularly large animal models. Nonetheless, gene modified large animal models are emerging and transgenic large animal models are likely to become a feasible option to auditory research in the very near future.

Finally, one key aspect for consideration is the ethics and cost associated with the maintenance of large or small animals for research purposes, including housing needs and resources required to maintain these animals. In common with use of any animals for research, full justification for their use is required and every available effort made to minimise numbers used and any suffering must be consistent with internationally accepted conventions stated in the ARRIVE guidelines [219]. With regard to ethical practice, challenges unique to use of the sheep relate to their body sizes and their natural behaviour. Body size of large animals obviously means that larger spaces are required to house animal adequately from practical perspective, but also to meet ethical, behavioural and welfare standards. The natural environment for sheep is the open field. An open pasture environment for breeding and keeping sheep is optimal. Our NZ institution has a research farm that meets husbandry standards that meet NZ’s legislative requirements and welfare codes for sheep [220]. In our care sheep that need to be maintained within an experiment for extended periods, can be returned from laboratory situations to research farm pasture. Furthermore, as sheep live in a flock social interaction is a very important aspect for their welfare. When road transport of sheep is required Institutional and National Welfare Codes of best practice for sheep husbandry stipulate that these social animals should be transported in small groups rather than individually. Generally there is a higher monetary cost for maintenance, feeding, breeding, surgery, and veterinary care compared to small rodent models [221].

In our experience in NZ, because we have a major primary industry centred around farming of sheep, we can acquire experimental subjects from large commercial populations on a regular basis. The cost of sheep for research of course depends on the local market at the time, currently ranging from NZ$250 to NZ$350 per ewe. This is compared to the cost of standard (non-genetically modified) rodents which range from NZ$60 to NZ$200 depending on the age. Additional costs for sheep work is the requirement to have a team of investigators capable of handling the animals, larger facilities required for surgeries and manipulation of the sheep, and larger volumes of reagents and medications. When live sheep work is undertaken in an urban laboratory setting the cost of can be around, 5–10 times higher (~ $3000) than the cost of performing equivalent experiment on rats (~ $300). However, our research farm laboratory facility and pasture are co-located which negates the problems of transporting animals long distances and also offers superior animal welfare standards and lower costs. In our experience, the use of large animals requires collaborative work with researchers with strong veterinary and animal husbandry backgrounds. Finally, every effort should made to share the use of valuable animals with other researchers who can use other tissues and samples. We hope this review will be helpful to those considering utility of large animal models and will support collaborative use of large animal models in the auditory research field.

## Conclusions

In this article, we compared some of the key features of the auditory system between different animal models. The shorter reproductive cycle and larger litter size makes small animals, such as mice, have an advantage over large animals for establishing the genetically modified hearing loss models. Large animals such as sheep have similar hearing range, adult and infant body size, and cochlear and round window membrane anatomy to humans and longer life span compared to other small animals. With similar cochlear geometry, perilymph volume (or ST volume), RWM thickness and diameter, and cochlear length to humans, sheep may be an ideal animal model for preclinical testing of new therapies, including diagnostic, drug delivery and prosthetic devices such as CI and other emerging technologies. Combined use of small animals for research that require higher throughput and genetic modification and large animals for medical translation will greatly accelerate the overall translation of basic research in the field of auditory neuroscience from bench to clinic.

## Data Availability

Raw data used for the figure presented in this article will be made available upon request to the corresponding author.

## Abbreviations

ARHL: Age-related hearing loss
AD: Alzheimer’s disease
ABR: Auditory brain response
CSF: Cerebrospinal fluid
CI: Cochlear implant
dBHL: Decibel hearing level
IHC: Inner hair cell
OoC: Organ of Corti
OAE: Otoacoustic Emissions
OHC: Outer hair cell
RW: Round window
RWM: Round window membrane
ST: Scala tympani
SNHL: Sensorineural hearing loss
SPL: Sound pressure level
SGN: Spiral ganglion neurons
