# Correction: Sheep as a large animal model for hearing research: comparison to common laboratory animals and humans

**DOI:** 10.1186/s42826-024-00217-3

**Published:** 2024-08-28

**Authors:** Po-Yi Lue, Mark H. Oliver, Michel Neef, Peter R. Thorne, Haruna Suzuki-Kerr

**Affiliations:** 1 Department of Physiology, The University of Auckland, Auckland, New Zealand; 2 Eisdell Moore Centre, The University of Auckland, Auckland, New Zealand; 3 Liggins Institute, The University of Auckland, Auckland, New Zealand; 4 Ngapouri Research Farm Laboratory, University of Auckland, Waiotapu, New Zealand; 5 Department of Surgery, Auckland District Health Board, Auckland, New Zealand; 6 Section of Audiology, The University of Auckland, Auckland, New Zealand

**Correction:** ***Lab Anim Res*** **39, 31 (2023)**

10.1186/s42826-023-00182-3

Following publication of the original article [1], the authors realized that one reference, [2], cited in [3] as the original source of sheep audiogram data had not been included.

A new reference “222. Wollack CH. (1963). The Auditory Acuity of the Sheep (Ovisaries). Princeton University” should be added to the reference list.

In Fig. 1, “Sheep [44]” has been corrected to “sheep [222]”.

In Table 2, the reference for Sheep species was originally “Heffner and Heffner [44]” and has been corrected to “Wollack [222]”.

The original article [1] has been updated.
